# Hyperprolactinemia in Functional Dyspepsia: The Entangled Domperidone Link

**DOI:** 10.7759/cureus.50927

**Published:** 2023-12-21

**Authors:** Devansh Bajaj, Mohamad Akram

**Affiliations:** 1 Gastroenterology, Jagadguru Sri Shivarathreeshwara (JSS) Medical College, Mysore, IND; 2 Internal Medicine, Swami Rama Himalayan University, Dehradun, IND

**Keywords:** comparative prolactin levels, prokinetic drugs, domperidone, functional dyspepsia, hyperprolactinemia

## Abstract

The commonest medications prescribed in functional dyspepsia are prokinetic agents, specifically domperidone. However, its administration at times elevates serum prolactin levels, which can lead to pathological hyperprolactinemia. The present study investigated the effect of 28 days of 30 mg domperidone therapy on prolactinemia in functional dyspepsia patients. We recruited 97 patients (60 men and 37 women, aged 18-80 years) who had functional dyspepsia diagnosed as per the Rome IV criteria. After taking a preliminary clinical history, we measured and compared serum prolactin levels at day ‘zero’ and day ‘twenty-eight’. We found increased prolactin levels from day '0' to day '28' after treatment with domperidone in functional dyspepsia patients, specifically in male participants aged less than 40 years, who are married and belong to middle socioeconomic status. The most common functional dyspepsia symptom found was pain in the epigastric region. To conclude, our pragmatic domperidone-induced-hyperprolactinemia link warrants this side effect to be robustly taken into account while treating functional dyspepsia patients with domperidone.

## Introduction

Functional dyspepsia, a term coined for recurring symptoms of an upset stomach, with no obvious cause is a common presenting symptom in general medical practice [1]. Approximately 25% of the population gets affected by dyspepsia every year, many of whom do not visit to seek medical advice. While one-fourth of them have some etiology, the remaining three-fourths remain idiopathic i.e., no cause can be detected on diagnostic evaluation [2-4]. The magnitude of dyspepsia among the general population is of great concern since patients who arrive at healthcare setups with indigestion symptoms are enormous, specifically an incidence of 20-30% worldwide [5] and 30.4% in the subcontinent [6]. Intriguingly, a study from India reported a 49% prevalence of irritable bowel syndrome [7]. Despite its unclear pathophysiology, various theories and mechanisms have been suggested. These mechanisms (based on dyspepsia sub-classification) differ from each other [8]. Furthermore, there is some evidence of association with motility disorders such as delayed and rapid gastric emptying, antral hypomotility, impaired gastric accommodation in response to meals, and gastric dysrhythmias [8-11]. However, these occur only in a subset of patients i.e. 25-35% experience dyspepsia with hypomotility, and only 10% have had rapid gastric emptying [12-14]. For a diagnosis of dyspepsia, symptoms must originate from the stomach and initial duodenum, specifically pain in the epigastrium which in conjecture with other related symptoms can be classified as functional dyspepsia. Notably, by the time dyspepsia is diagnosed, by endoscopy, most patients experience some form of dyspeptic symptoms [15].

Although the commonest medications prescribed in functional dyspepsia are antacids, in most patients with symptoms of gastroduodenal dysmotility (bloating, early satiety), inhibitors of proton pump and prokinetic agents, such as metoclopramide, domperidone, mosapride, itopride, and levosulpiride, are the mainstay of treatment for functional dyspepsia [16]. Importantly, as per the management plan of functional dyspepsia, both in the Asian subcontinent and the United States of America, there is guidance related to changing to a drug other than a prokinetic or proton pump inhibitor if adequate efficacy has not been achieved four weeks after treatment [16]. Domperidone is a dopamine receptor antagonist acting on dopamine II receptors found intracranially and extracranially [17]. The prokinetic action of domperidone is largely attained via intensified motility of the esophagus which amplifies the contraction of the antral and duodenal regions [17]. It improves the peristaltic coordination across the pylorus and accelerates the gastric content emptying [18-20]. Contrarily, prolactin, a polypeptide hormone released from the anterior pituitary, is mainly responsible for lactation and breast development; however, it has a myriad of other actions needed to maintain homeostasis including osmoregulation, growth, reproduction, integument, and synergism with corticosteroids [21], effects on growth and development, water and electrolyte balance, endocrinology and metabolism, brain, and behavior and immunoregulation [22].

Most intriguingly, domperidone, the aforementioned drug used for treating dyspepsia, can sometimes result in pathological hyperprolactinemia [23]. Domperidone stimulates prolactin secretion potentially via dopaminergic mechanism by inhibiting the prolactin inhibiting factor (PIF) at the level of hypothalamus, consequently fostering uninhibited prolactin release i.e., hyperprolactinemia. In a study conducted by Cho et al., the prolactin level was monitored in patients with cystic fibrosis receiving domperidone, almost 45 percent of the patients had values above the normal range (<20 ng/dl), and dosage adjustment or discontinuation was required in only 14 percent of patients with raised prolactin levels [24]. Another study conducted by Kaufman et al. reported that oral domperidone caused sustained elevation of serum prolactin levels in patients with an autonomous goiter, i.e., the response to domperidone (on prolactin and thyroid stimulating hormone levels) was higher than what was caused by giving intravenous TRH [25]. Thus, to explore this intriguing link between domperidone and prolactin, the present study was undertaken by investigating the effect of 30 mg domperidone therapy (for 28 days) on prolactinemia in functional dyspepsia patients.

## Materials and methods

Participants

This study, designed as a cross‑sectional-observational research, was reported per the Strengthening the Reporting of Observational Studies in Epidemiology (STROBE) guidelines for reporting observational studies. From June 2019 to June 2020, we recruited 97 patients (60 men and 37 women, aged 18-80 years) with functional dyspepsia (without alarm symptoms) visiting the Department of Medicine, Himalayan Institute of Medical Sciences (HIMS), Swami Ram Nagar, Dehradun, India. Inclusion criteria were adults (> 18 years, males and females), primary diagnosis of functional dyspepsia, and the absence of alarming symptoms. Exclusion criteria were patients with alarming symptoms, patients on any drugs known to cause functional dyspepsia, and pregnant and lactating women. The HIMS Research and Ethics Committee approved this study before patient enrolment, with each of them voluntarily consenting in writing for participation.

Study protocol and tools

ROME IV criteria were used to provisionally diagnose functional dyspepsia. First, written and informed consent was taken, and then a comprehensive history related to the associated symptoms was taken, followed by a personal history, specifically related to illicit drug addiction in conjunction with associated concomitant complaints. Female patients were inquired in detail about menstrual history, contraception, breastfeeding, and recent mammography scans. A thorough general physical examination was performed thereafter before patients were briefed about the provisional diagnosis of functional dyspepsia (Rome IV criteria). Furthermore, intake of domperidone, 30 mg (OD: once a day), was instructed, and serum samples were collected for prolactinemia, thyroid stimulating hormone, creatinine, alanine transferase, and hemoglobin (except prolactinemia, most were optional). The domperidone medications lasted for 28 days. On the last day of their visit, after ensuring treatment compliance by history, a serum sample (prolactinemia) was drawn again to contrast its concentration on the first day zero and the last day (end of four weeks) to infer accordingly. While standardized tools were instituted to collect the demographic and the clinical data, prolactinemia was quantitatively assessed with the serum Prolactin (PRL) ELISA kit with the least PRL concentration hovering at 2 ng/ml, approximately.

Sample size

The sample size estimation formula, n = Z2α/2p/l2, was used to calculate the sample size: (n) is the required sample size, p prevalence of prolactinemia in functional dyspepsia (50%), α is the level of significance, taken as 5%, and ‘I’ is the relative error - 20% of prevalence (p) to estimate sample size which gives n=97. The HIMS Research and Ethics Committee gave erstwhile approval for this study (Ref. No: SHU/HIMS/RC/2023/277). Additionally, all the patients gave their verbal consent prior to sample collection.

Statistical analysis

 IBM SPSS Statistics for Windows, Version 19 (Released 2010; IBM Corp., Armonk, New York, United States) was used for data and correlational analysis throughout this study. The demographic variables were investigated, under the unified lens of continuous and categorical variables. Categorical variables were presented as frequency and percentage, whereas mean and standard deviation described the most continuous variables. Furthermore, the mean difference was tested, using the Pearson Chi-square test, with a p-value < 0.05, conventionally considered statistically significant.

## Results

Participants demographics

Overall, the mean age of the participants was 45.85 + 16.50 years (range 18-80 years). Most recruited patients aged <40 years (41.2%), which reduced chronologically to 41-60 years (36.1%) and 61-80 years (22.7%). Men comprised a vast majority (61.9%), leaving females numbered 38.1%. Details of participant characteristics are presented in Table 1.

**Table 1:** Demographic characteristics of patients with functional dyspepsia Reference range of prolactin: Less than 20 ng/mL (425 µg/L)

Demographic Characteristics	Frequency	Percentage (%)
Age (years)		
<40	40	41.2
41-60	35	36.1
61-80	22	22.7
Gender		
Male	60	61.9
Female	37	38.1
Marital Status		
Married	86	88.7
Unmarried	11	11.3
Socioeconomic status		
High Class	11	11.3
Middle Class	75	77.3
Lower Class	11	11.3
Bothersome postprandial fullness		
Yes	45	46.4
No	52	53.6
Bothersome early satiety		
Yes	43	44.3
No	54	55.7
Bothersome epigastric pain		
Yes	61	62.9
No	36	37.1
Bothersome epigastric burning		
Yes	57	58.8
No	40	41.2
Past clinical history		
Hypertension	28	28.9
Diabetes mellitus	28	28.9
Thyroid disorder	0	0.0
Chronic Kidney Disease	2	2.1
Dietary habits		
Mixed	66	68.0
Veg	31	32.0
Bowel & bladder habits		
Constipation	31	32.0
Increased Urinary Frequency	1	1.0
Personal habits		
Smoking	19	19.6
Alcohol	14	14.4
Marijuana	1	1.0
Tobacco	1	1.0
Prolactin-Day 0 (ng/mL)		
<20	59	60.8
>/=20	38	39.2
Prolactin- Day 28 (ng/mL)		
<20	19	19.6
>/=20	78	80.4

Comparative prolactin levels at day 0 and day 28

Table 2 depicts the comparative distribution (frequency & %) of patients, based on their prolactin levels - at day ‘0’ and day ‘28’. On day 0, prolactin levels were less than 20 (ng/mL) in 60.8% of patients and more than 20 in 39.2% of patients. However, on day 28, prolactin levels were less than 20 (ng/mL) in 19.6% of patients and more than 20 in 80.4% of patients.

**Table 2:** Comparative distribution (frequency & %) of patients based on their prolactin levels at day 0 and day 28 Reference range of prolactin: Less than 20 ng/mL (425 µg/L)

	Prolactin levels (ng/dl) at Day 0; Frequency (%)		Prolactin levels (ng/dl) at Day 28; Frequency (%)		p-value
	<20	≥20	<20	≥20	
	59 (60.8)	38(39.2)	19(19.6)	78(80.4)	<0.001
Total	97(100)		97(100)		

Association between demographics and serum prolactin levels at day 28

Table 3 presents the association between demographic characteristics and serum prolactin levels at day 28. Patients with elevated prolactinemia at the end of four weeks were in the age range below 40 years (41%); trailed by 41-60 years (33.3%) and 61-80 years (25.6%) patients. There were males (57.7%) and females (42.3%), and they were mostly married (87.2%). The majority belonged to the middle socioeconomic status (80.8%), with a few highs (10.3%) and lower socioeconomic status (9%), respectively. The most bothersome symptoms include postprandial fullness (35/45), early satiety (32/43), epigastric pain (53/61), and epigastric burning (50/57). Altogether, the associations of age, sex, marital status, socioeconomic status, postprandial fullness, and early satiety with the prolactin levels at day 28 were found to be statistically not significant, except tenderness (p=0.04) and burning sensations in the epigastrium (p=0.02).

**Table 3:** Association between demographic characteristics and serum prolactin levels at day 28

Demographic Characteristics	Prolactin – Day 28		Chi-Square	p-value
	<20 (ng/mL)	≥20 (ng/mL)		
	Frequency (%)	Frequency (%)		
Age (years)				
<40	8 (42.1)	32 (41.0)		0.30
41-60	9 (47.4)	26 (33.3)	2.37	
61-80	2 (10.5)	20 (25.6)		
Gender				
Male	15 (78.9)	45 (57.7)	2.92	0.07
Female	4 (21.1)	33 (42.3)		
Marital Status				
Married	18 (97.4)	68 (87.2)	0.86	0.32
Unmarried	1 (5.3)	10 (12.8)		
Socioeconomic status				
High Class	3 (15.8)	8 (10.3)		0.22
Middle Class	12 (63.2)	63 (80.8)	2.99	
Lower Class	4 (21.1)	7 (9.0)		
Bothersome post prandial fullness				
Yes	10 (52.6)	35 (44.9)	0.37	0.36
No	9 (47.4)	43 (55.1)		
Bothersome early satiety				
Yes	11 (57.9)	32 (41.0)	1.76	0.41
No	8 (42.1)	46 (59.0)		
Bothersome epigastric pain				
Yes	8 (42.1)	53 (67.9)	4.37	0.04
No	11 (57.9)	25 (32.1)		
Bothersome epigastric burning				
Yes	7 (36.8)	50 (64.1)	4.68	0.02
No	12 (63.2)	28 (35.9)		

## Discussion

The primary objective of this study was to explore the effect of four weeks of empirical domperidone therapy on prolactinemia in patients with functional dyspepsia. Our results showed that except for a borderline significant association for epigastric tenderness (p=0.04) and burning (p=0.02), none of the remaining demographic characteristics were associated with elevated 28-day prolactin levels (≥20 (ng/mL).

Comparison to literature

In our study, patients with elevated prolactinemia at the end of four weeks were in the age range below 40 years (41%) trailed by 41-60 years (33.3%) and 61-80 years (25.6%) patients (Table 3). The complaints of functional dyspepsia were higher in patients ≤60 years of age, and very few patients ≥60 years of age came to OPD for dyspepsia. Our findings were in accordance with those of Jain et al., who contrasted the efficacy of two different varieties of gastric motility drugs treating functional dyspepsia. The majority of his patients were aged 20-40 years (69%), and only 15% of patients ≥60 years of age [26]. Similarly, Narayanan et al., in their study on the efficacy and acceptability of acotiamide as a gastric motility drug in functional dyspepsia patients, reported more than 50% of the patients who have had frequent visits to the outdoor department who complained of dyspepsia aged 40-60 years [27]. In our study, 61.9% of men and 38.1% of women comprise the overall 97 patients (Table 1). Congruent to ours, Jain et al. observed 122 male patients and 49 female patients, with a male preponderance (71.35%) in his sample group [26]. However, a few studies hinted at the heightened shift of functional dyspepsia toward the female sex, conflicting with what we found, which is in accord with the study by Jones et al. across the United Kingdom, who reported dyspeptic female preponderance [28].

We further found ‘bothersome postprandial fullness’ and ‘bothersome early satiety’ present in 46.4% and 44.3% of our patients respectively, and ‘bothersome epigastric pain’ and ‘bothersome epigastric burning’ in 62.9% and 58.8% patients, respectively (Table 1). These findings are in stark contrast to some of the studies out there. For instance, Jain et al. reported that a major chunk of his patients complained of stomach pain (42.3%) bloating (88.8%), nausea (74.8%), and fullness as a myriad of symptoms in greater chunks in his patients. However, blenching (7%) along with regurgitation (12.9%) forms a smaller bit of the overall symptomatology [26]. Additional work by Zagari et al., reported similar findings in his cohort of 114 dyspeptic patients, although his key dyspeptic symptom intriguingly turns out to be postprandial fullness (67%) [29]. We are also in close concordance with Matsueda et al., who also investigated and reported postprandial fullness in more than half of his 405-patient group [30]. In a nutshell, postprandial fullness emerges to be the standout symptom for most dyspepsia patients.

**Figure 1:**
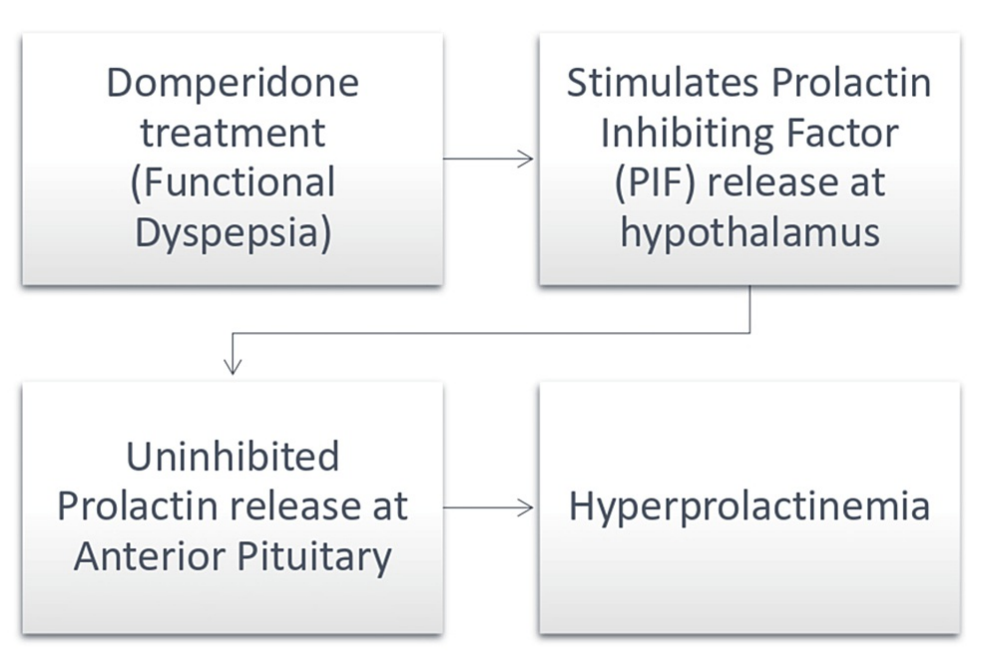
Proposed association between elevated prolactin levels and domperidone therapy in functional dyspepsia Image credits: Mohamad Akram

We found prolactinemia <20 ng/dl in about 3/5th of our patients whilst >20 ng/dl in almost 2/5th on day ‘zero’. However, this trend significantly reversed on day 28 when we had just 1/5th of patients with prolactinemia <20 and a whopping 4/5th with >20 ng/dl values (Table 1). Hyperprolactinemia on day 28 was present in 53.8% of patients in which prolactin levels were <20 ng/ml on day 0 and in 46.2% of patients in which prolactin levels were >/=20 ng/ml on day '0' (Table 3). There was hyperprolactinemia on day '28', regardless of the day ‘zero’ prolactinemia concentration. A myriad of studies worldwide reported similar findings. E.g., Fujino et al., reported credible efficacy of domperidone in prolactinemia [31]. We found our results similar to Vilar et al., who also reported hyperprolactinemia via domperidone administration [32], and akin to the findings of our study, Cho et al. also found hyperprolactinemia in domperidone-treated patients as his patients showed to the hospital [24]. Further evidence of hyperprolactinemia-induced oligomenorrhea was also reported in yet another young female patient who was undergoing domperidone therapy for dyspeptic symptoms [23]. Overall, we feel we have a strong evidence-based rationale and proposed association/mechanistic hypothesis (Figure 1) to believe that hyperprolactinemia is an aftermath of domperidone therapy, specifically in women.

Strengths

First, our robust longitudinal design edges our results over most cross-sectional contemporary trials. Second, the use of Rome IV criteria helped us pinpoint functional dyspepsia diagnosis accurately. Third, we assessed prolactinemia quite accurately with the serum Prolactin (PRL) ELISA kit.

Limitations

Diverse causes of hyperprolactinemia were not ruled out. While a history of thyroid disorder was considered, individual thyroid profiles for each patient could not be tested. The organic cause of dyspepsia was not scrutinized in every patient. Clinical features of hyperprolactinemia were not studied in correlation with its level of elevation i.e., mild, moderate, or severe. Our small sample size (n=97) limits our findings. Thus, the inclusion of a larger cohort may reveal more conclusive findings. Since most comparative studies are from the Asian subcontinent, it would constrain the generalizability of our results, specifically in the Western population (Europe and North America).

## Conclusions

Functional dyspepsia is a widespread and worrisome clinical entity that has been managed safely with a myriad of gastric motility agents, specifically domperidone. While other side effects including disorientation, dizziness, lightheadedness, etc. are reported, hyperprolactinemia is a prominent one. An overlooked domperidone-induced-hyperprolactinemia diagnosis may result in unwarranted investigations that can cost patients a fortune. After taking relevant clinical history and examination, serum prolactin levels at day ‘zero’ and day ‘twenty-eight’ were measured and equated. We found increased prolactin levels from day 0 to day 28 after treatment with domperidone in functional dyspepsia patients, specifically in males aged less than 40 years, who are married and belong to middle socioeconomic status. Our pragmatic domperidone-induced-hyperprolactinemia link warrants this side effect to be robustly taken into account while treating functional dyspepsia patients with domperidone. Physicians should look for alternative hypermotility agents to treat functional dyspepsia, other than domperidone, specifically in female patients. Also, reaching a definitive diagnosis avoids unnecessary brain imaging in hyperprolactinemia patients.
